# A corpus for plant-chemical relationships in the biomedical domain

**DOI:** 10.1186/s12859-016-1249-5

**Published:** 2016-09-20

**Authors:** Wonjun Choi, Baeksoo Kim, Hyejin Cho, Doheon Lee, Hyunju Lee

**Affiliations:** 1 School of Information and Communications, Gwangju Institute of Science and Technology, Chemdangwagi-ro, Gwangju, Republic of Korea; 2 Department of Bio and Brain Engineering, KAIST, Yuseong-gu, Daejeon, Republic of Korea

**Keywords:** Data mining, Text mining, Natural product, Plant, Chemical, Corpus, Natural language processing, Medicine

## Abstract

**Background:**

Plants are natural products that humans consume in various ways including food and medicine. They have a long empirical history of treating diseases with relatively few side effects. Based on these strengths, many studies have been performed to verify the effectiveness of plants in treating diseases. It is crucial to understand the chemicals contained in plants because these chemicals can regulate activities of proteins that are key factors in causing diseases. With the accumulation of a large volume of biomedical literature in various databases such as PubMed, it is possible to automatically extract relationships between plants and chemicals in a large-scale way if we apply a text mining approach. A cornerstone of achieving this task is a corpus of relationships between plants and chemicals.

**Results:**

In this study, we first constructed a corpus for plant and chemical entities and for the relationships between them. The corpus contains 267 plant entities, 475 chemical entities, and 1,007 plant–chemical relationships (550 and 457 positive and negative relationships, respectively), which are drawn from 377 sentences in 245 PubMed abstracts. Inter-annotator agreement scores for the corpus among three annotators were measured. The simple percent agreement scores for entities and trigger words for the relationships were 99.6 and 94.8 %, respectively, and the overall kappa score for the classification of positive and negative relationships was 79.8 %. We also developed a rule-based model to automatically extract such plant–chemical relationships. When we evaluated the rule-based model using the corpus and randomly selected biomedical articles, overall F-scores of 68.0 and 61.8 % were achieved, respectively.

**Conclusion:**

We expect that the corpus for plant–chemical relationships will be a useful resource for enhancing plant research. The corpus is available at http://combio.gist.ac.kr/plantchemicalcorpus.

## Background

Plants are a type of natural product that includes trees, herbs, edible foods, among others [1]. They are known to be abundant sources of chemicals with potential therapeutic effects [2]. Furthermore, since natural compounds have been empirically proven to have relatively fewer side effects and unwanted reactions, plants have been widely used for thousands of years for the treatment of diverse diseases and their symptoms [3]. Because of these advantages of plants, many studies have been carried out assessing the effectiveness of plants against diseases [4–6], and the number of patents related to pharmaceutical natural products including plants has continuously increased [7]. To further enhance such efforts, identification of active substances or chemical compounds in plants is important because many diseases can be relieved or treated by chemicals in plants that control the activities of proteins related to diseases [8, 9].

In this respect, many researchers have tried to construct public databases containing plant-related information, especially plant–chemical relationships that represent which compounds are included in which plants. In general, such data were manually collected from books, published results, and empirically widely known facts. One of the most representative databases containing plant–chemical relationships is the traditional Chinese medicine (TCM) database@Taiwan [10], which is a 3D small molecular structure database of TCM for virtual screening or molecular simulation. Although this database currently contains 32,364 compounds from 352 different herbs, animal products, and minerals, which were manually collected from medical texts and scientific publications, only a small fraction of plants used for medicinal purposes are included in the database. TCMID [11] is one of the largest TCM databases providing TCM-related data, including prescriptions, herbs, herbal ingredients, targets, drugs, diseases, and relationships between these entities. This database was assembled by applying text mining methods to biomedical articles and by integrating other public databases such as TCM-ID [12], TCM database@Taiwan [10], HIT [13], STITCH [14], OMIM [15], and DrugBank [16]. However, even in TCMID, only 8159 plants (referred to as “herb” in TCMID) are currently provided, which is relatively small compared to the more than 150,000 plants defined in the NCBI Taxonomy database [17].

With the continuous accumulation of biomedical articles, it is possible to extract such plant–chemical relationships from the literature if proper corpora and text mining (TM) models are available. Until now, few TM systems that extract information about chemicals contained in plants from biomedical articles have been developed [18]. Jensen et al. [18] proposed an integrated text mining system based on manually constructed corpora for analyzing associations between plants and health effects. They extracted plant–chemical and plant–disease relationships from biomedical articles using text mining models and then integrated these two relationships to infer chemical–disease relationships. Although Jensen et al. [18] has presented a text mining model to extract plant–chemical relationship, a corpus used to text-mine the relationship is not available for public use. Generally, developing a corpus requires significant efforts because several annotators need to manually identify entity names, trigger words, and positive or negative relationships in the articles. Because most text mining methods require an annotated corpus to learn models for detecting entity mentions and for extracting relationships between entities, providing a corpus specific for each domain is important [19]. Therefore, a plant–chemical corpus with a proper format such as the BioC XML format [20], which is a common interchange format widely used in the BioNLP community, is important for constructing and evaluating future text mining systems that extract plant–chemical relationships from texts.

Our study aims to develop a corpus for plant–chemical relationships. Here we describe processes for constructing the corpus for two entities of plants and chemicals and their plant–chemical relationship. In addition, we construct and evaluate a rule-based model, which automatically extracts plant–chemical relationships from articles. In this work, “plant–chemical relationships” are classified into two types: (i) a positive relationship means that a plant contains a chemical, i.e., a chemical is derived from a plant or a chemical is a part of the molecular structure of a plant (e.g., ***actinidin**** has previously been reported as the major allergen in ****kiwifruit***); (ii) a negative relationship means that there is no information specifying that a plant contains a chemical (e.g., *both****parthenolideandFeverfew extract showed a time-dependency in their action***). The corpus currently consists of 267 plant entities, 475 chemical entities, and 1,007 plant–chemical relationships (550 and 457 positive and negative relationships, respectively), which are drawn from 377 sentences in 245 PubMed abstracts. Our corpus will be useful for developing new natural language processing (NLP) tools related to plant–chemical relationships.

## Related works

Automatic extraction of semantic relationships between domain-specific entities from articles requires recognition of the entity names and syntactic analysis of texts. For this task, an annotated corpus is necessary. Thus, we review several corpora and text mining systems related to chemicals and plants.

The Linnaeus corpus [21] annotates entities related to species and organisms, including plants, from 100 full-text articles in the PMC Open Access document data set. It was built for tools to recognize and to normalize species names and uses a dictionary-based approach with the NCBI taxonomy data. The Species corpus [22] remedies the shortcomings of the Linnaeus corpus, which annotates entities at the full-text level, by annotating entities at the abstract level to increase variability of species names. They selected 100 abstracts from journals in the following eight categories: bacteriology, botany, entomology, medicine, mycology, protistology, virology, and zoology. The corpus currently contains a total of 800 abstracts with annotated information of species mentions. The corpus was used for the development and evaluation of their NER tool based on the dictionary provided by the NCBI taxonomy database [17] for detecting species names.

The CHEMDNER corpus [23] is the most comprehensive data for the development of named entity recognition (NER) systems in the chemical domain. It contains a total of 84,355 chemical entity names from 10,000 PubMed abstracts, which were manually annotated by expert chemistry curators. Each abstract was carefully selected based on document selection criteria to be representative of a wide range of chemistry-related fields. Each chemical entity names was assigned to one of the following seven different subtypes: abbreviation, family, formula, identifier, multiple, systematic, and trivial. The authors of the corpus also provided detailed guidelines for identifying entity names with a proper entity class. The CHEMDNER corpus and the proposed annotation guidelines, which can be expanded by users, are publicly available so that they can be used for researchers developing TM systems in the area of chemicals. However, the corpus does not contain any relationship information.

Li et al. [24] performed manual annotation of chemical and disease entities and relationships between them for the BioCreative V challenge of recognizing disease name entities and extracting chemical-induced disease relationship. The corpus in the BioC XML format currently contains 1,500 PubMed articles with 4,409 chemicals, 5,818 diseases, and 3,116 chemical–disease interactions, all manually annotated. They used some annotation tools including PubTator [25] and NER tools such as DNorm [26] and tmChem [27] to accelerate manual annotation. The strength of this corpus is that chemical–disease relationships were extracted from both within a sentence and across sentence boundaries. Along with [23, 24], there are several chemical- or drug-related corpora such as Comparative Toxicogenomics Database corpus [28] and a corpus contained in the Pharmspresso database [29]. However, there are so far no publicly available corpora for plant–chemical relationships.

Jensen et al. [18] proposed an integrated text mining approach and chemoinformatics analysis to enhance understanding of how plant-based diets (fruits, vegetables, and plant-based beverages) affect human health and disease prevention. They accumulated 369,549 plant–phytochemical edges from 23,137 compounds and 15,722 plants and 38,090 plant–disease associations from 7,178 plants and 1,613 human disease phenotypes using a Naive Bayes classifier. These relationships were extracted from 21 million PubMed abstracts. Using chemical–disease relationships inferred from text-mined relationships, they applied chemoinformatics methods to analyze the molecular-level association of a plant-based diet to diseases. For the development and evaluation of the text mining model, an in-house corpus for each relationship type was also constructed. However, the corpus is not publicly available.

## Methods

### Sentence collection and preprocessing

**Fig. 1:**
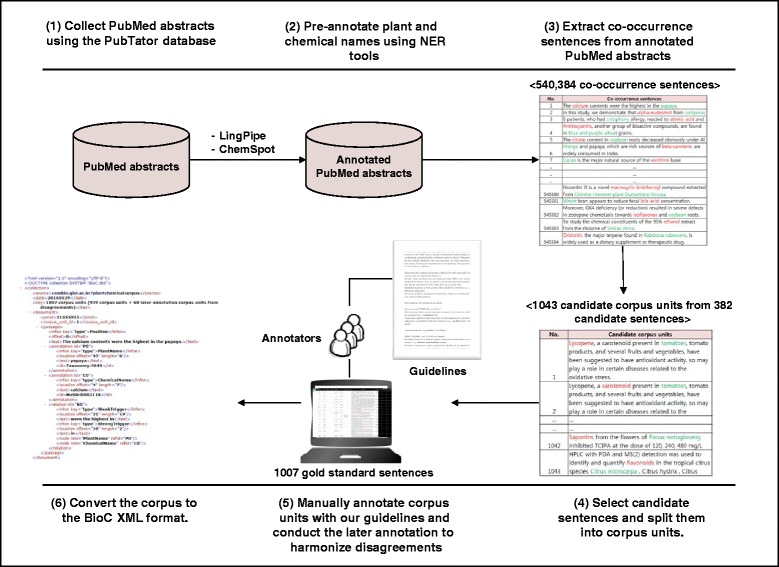
The workflow of corpus construction. The corpus was constructed as follows: (i) we collected PubMed abstracts from the PubTator database; (ii) we applied NER tools, including LingPipe and ChemSpot, to pre-annotate plant and chemical names; (iii) we extracted co-occurrence sentences that contain at least one plant and chemical name; (iv) we randomly selected candidate sentences, where the numbers of positive and negative sentences were set to be approximately the same, and also split them into corpus units; (v) we manually annotated candidate corpus units with our guidelines and also conducted later annotation to harmonize disagreements after annotators finished their annotation tasks; and (vi) we converted annotated corpus units to the BioC XML format

This section describes selection of candidate sentences before constructing the corpus as shown in Fig. 1. For this task, we automatically extracted 13,408,621 PubMed abstracts using PubTator [25], which provides functions for users to download all PubMed abstracts. Then, the following preprocessing steps were performed on the collected PubMed abstracts.

**Fig. 2:**
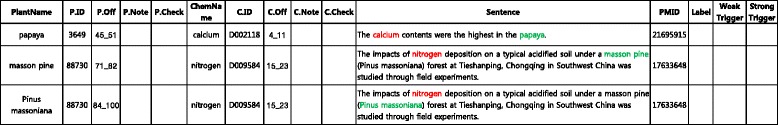
Annotation example in Excel file. The candidate corpus units were exported in Excel format so that annotators could easily annotate the corpus. The first and second columns represent annotated plant names and IDs contained in each sentence shown in the eleventh column. The sixth and seventh columns show annotated chemical names and IDs. Annotators should check whether plant and chemical names and their IDs are correctly annotated. If they are incorrectly annotated, then annotators should write the letter “X” in the fifth, tenth, or both columns. Also, they should leave comments in the fourth, ninth, or both columns. For the thirteenth column, annotators should determine whether the relationship in the sentence is positive or negative. If the sentence contains a positive relationship, the weak and strong triggers should be written in the last two columns

1. For pre-annotating plant names in abstracts, a plant name dictionary was first constructed using public data from TCMID [11] and NCBI Taxonomy [17]. The dictionary contains 333,686 plant names in English, Chinese, and Latin and it is available to download at our corpus web site. After constructing the dictionary, LingPipe [30], a dictionary-based exact-matching NER tool, was applied to all collected abstracts to locate plant names. Chemical names were annotated using ChemSpot [31], which is a specialized tool for locating chemical names that covers trivial names, drugs, abbreviations, and molecular formulas in texts. For the annotation of chemical identifiers (IDs), three types of IDs were used: MeSH, CHEMBL, and CAS Registry Number.
2. Using pre-annotated abstracts from step one, we collected 540,384 co-occurrence sentences in which at least one plant name and at least one chemical name co-occur. The rest of the sentences that do not contain either a plant name or a chemical name were excluded.
3. In this step, the main annotator selected, from 540,384 co-occurrence sentences, candidate sentences to be manually annotated by annotators in the “Annotating the corpus” step. Of the co-occurrence sentences, the number showing a negative relationship was larger than the number showing a positive relationship. Because positive relationships are more informative than negative relationships for showing that a plant contains a chemical, we constructed balanced numbers of positive and negative relationships. Hence, the main annotator randomly selected candidate sentences from the co-occurrence sentences and manually classified each sentence into positive or negative classes, where the numbers of positive and negative sentences were set to be approximately the same. In addition, the main annotator validated whether all the entity mentions and their IDs in sentences were correctly annotated by NER tools. If contents such as mentions and IDs were incorrectly annotated, then the main annotator manually corrected them. When multiple pairs of plant and chemical names were found in a candidate sentence, each plant–chemical pair was classified into a positive or negative relationship; we call each pair “a corpus unit,” because more than one relationship can be found in a candidate sentence. For example, in the third line in Fig. 2, the candidate sentence has one chemical name and two plant names, which can produce two plant–chemical pairs (nitrogen–masson pine and nitrogen–Pinus massoniana). In this case, two different candidate corpus units were created from a single sentence (e.g., the third and fourth line in Fig. 2). Likewise, we split all candidate sentences into candidate corpus units.

In contrast with other existing corpora [23, 24], which annotate relationships at the abstract level, we collected the relationships at the sentence level using the above steps because we aimed to expand the diversity of plant names. In many plant-related abstracts or documents, the same plant terms appear multiple times, which may affect the quality of the corpus.

The candidate corpus units from candidate sentences selected in the sentence collection and preprocessing step were exported to an Excel format as described in Fig. 2 and then annotated by annotators according to the annotation guidelines described in the next subsection.

### Annotation guidelines

Annotation guidelines are defined to support validating NER results extracted by NER tools for entity names and to determine class labels of plant–chemical relationships during the annotation process. The guidelines were continuously updated by the main annotator during the sentence collection and preprocessing step. Because annotators need to check both NER results and relationships between two entities, the guidelines were categorized for the entity annotation and the relationship annotation.

#### Guidelines for entity annotation

Entity names in the corpus were first annotated by NER tools. However, due to inaccuracy of the NER tools, a fraction of annotated names might not be plants or chemicals, while actual plants or chemicals might be missed. Although these mistakes were initially checked by the main annotator, the other annotators also examined entities of plants and chemicals based on the guidelines.

The general guidelines for both entities are as follows:

- Annotators only examine a plant and a chemical for each corpus unit, colored in green and red, respectively (Fig. 2).
- Because entity names were pre-annotated using NER tools, annotators should check whether there are missed or incorrectly annotated names and IDs. IDs are annotated as “NA” when there are no proper IDs.
- Acronyms should be annotated. When it is not clear whether an acronym indicates a plant or chemical name, an annotator annotates an acronym if original long words are found in the corresponding PubMed abstract.

For plant names, the guidelines are as follows:

- Taxonomy and TCMID IDs are allowed.
- Plant names whose actual contextual meaning do not represent plants should not be annotated (e.g., ***cinnamon****rat*).
- Plant names include specific plants and a family of plants.
- Unnecessary adjectives and nouns next to plant names should not be annotated unless they are included in the plant dictionary (e.g., ***mashed****potato, tobacco****yield***).

For chemical names, the guideline is as follows:

- MeSH, CHEMBL, and CAS IDs are allowed.
- If multiple chemicals are linked together, annotators should consider them as a single name (e.g., ***linoleic and linolenic acids***).
- Receptors, transporters, genes, and proteins should not be annotated as a chemical name (e.g., ***chlorophyll-protein, tricarboxylate transporter, acetylcholine receptor***).

#### Guidelines for relationship annotation

Annotators need to annotate positive and negative relationships between plants and chemicals, which are defined as follows:

- Positive relationship: indicates that a plant contains a chemical; a chemical is derived from a plant, or the chemical is part of the molecular structure of the plant (e.g., ***Bilobalide**** (BB) is a sesquiterpenoid extracted from ****Ginkgo biloba****leaves*). In the example, we find a positive relationship between Ginkgo biloba (plant) and Bilobalide (chemical).
- Negative relationship: indicates that a corpus unit does not specify that a plant contains a chemical (e.g., *The****fenamiphostreatment outperformed all fosthiazate treatments in tobacco yield and root gall reduction***).

The guidelines for the relationship annotation are as follows:

- Annotators classify a sentence containing a plant and a chemical into a positive or negative relationship.
- When a chemical name contextually indicates one of the extraction solvents for plant extracts, annotators should classify this case as “negative relationship.”
  – “*[****methanol/ethanol/petroleum/chloroform/ isopropanol****]*_*chemical*_*extracts of [****ginger****]*_*plant*_”
- Metabolism is a process in a set of chemical reactions that modifies a chemical molecule into another molecule for storage or for immediate use in another reaction. According to the definition of metabolism, if a sentence represents that more than one chemical is involved in the metabolism process of a plant, annotators should regard all of them as ingredients of the plant.
  - “*[****26-Norbrassinolide****]*_*chemical*_*, identified as a ****metabolite of**** brassinolide in cultured cells of the [****liverwort, Marchantia polymorpha****]*_*plant*_*, as well as 26-norcastasterone and 26-nor-6-deoxocastasterone were synthesized*”
- Synthesis is a process that produces an organic compound in living things. By the definition of synthesis, if a sentence indicates a synthesis phenomenon between a plant and a chemical, annotators should regard this case as an ingredient of a plant.
  - “***Synthesis of****[****O-acylhomoserine esters****]*_*chemical*_* was detected only in [****Pisum sativum L****]*_*plant*_”
- If a sentence explains the positive relationship between a derived plant from an original plant and a chemical, annotators should consider that both the derived plant and the original plant contain the chemical.
  - “*[****Snapdragon****]*_*plant*_*in tomato****contains****[****anthocyanin****]*_*chemical*_”
  - “*Snapdragon in [****tomato****]*_*plant*_***contains****[****anthocyanin****]*_*chemical*_”
- Annotators should consider grammatical structures for deciding relationship types. In the example sentence below, there are two chemical names (anthocyanin) and three plant names (tomato, blackberries, and blueberries). As positive relationships, the former anthocyanin belongs to tomato and the latter anthocyanin belongs to blackberries and blueberries.
  - “*[****Anthocyanin****]*_*chemical*_***accumulation in****[****tomato****]*_*plant*_*and at concentrations comparable to the anthocyanin levels found in blackberries and blueberries*”
  - “*Anthocyanin accumulation in tomato and at concentrations comparable to the [****anthocyanin****]*_*chemical*_*levels****found in****[****blackberries****]*_*plant*_*and blueberries*”
  - “*Anthocyanin accumulation in tomato and at concentrations comparable to the [****anthocyanin****]*_*chemical*_*levels****found in****blackberries and [****blueberries****]*_*plant*_”
- Annotators should annotate both a weak trigger term and a strong trigger term when a corpus unit has a positive relationship. The weak trigger can be more than one term representing a plant–chemical relationship. On the other hand, the strong trigger should be a single word that is thought to be the most representative word explaining a plant–chemical relationship. In the example of the sentence “*The [****calcium****]*_*chemical*_*contents****were highest in****the [****papaya****]*_*plant*_,” “were highest in” and “in” are the weak trigger term and the strong trigger term, respectively.

### Annotating the corpus

This subsection describes the annotation process for plants, chemicals, and their relationships in all the candidate corpus units as shown in Fig. 1. The annotation task for the corpus was performed by three annotators with a basic knowledge of biology and traditional Chinese medicine. The main annotator performed sentence collection and preprocessing, built the annotation guidelines, and carried out the first annotation of the corpus. Two assistant annotators participated in the corpus annotation.

The corpus annotation work went through two phases (phases 1 and 2) because we divided the three annotators into two groups (Group 1: main annotator-assistant annotator 1, Group 2: main annotator-assistant annotator 2). Based on the guidelines, annotators in Group 1 and Group 2 manually annotated the different sets of candidate corpus units. A corpus constructed through the two phases is the main corpus in our work and is called “the primary corpus” hereafter to distinguish it from an additional corpus developed as described in the next section, Constructing a rule development corpus and a rule-based model. The annotation task was conducted by manually filling in fields of the Excel file as shown in Fig. 2, where “PlantName,” “P.ID,” and “P.Off” represent plant name, its identifier, and offset of the plant name in the text, respectively, and “ChemName,” “C.ID,” and “C.Off” represent chemical name, its identifier, and offset of the chemical name in the text. Details about other fields are also explained as follows:

- P.Check: Write the letter “O” when all of the contents in “PlantName,” “P.ID,” and “P.Off” shown in Fig. 2 are correctly annotated and write the letter “X” when any of them are incorrectly annotated.
- P.Note: Leave comments about incorrectly annotated contents in “PlantName,” “P.ID,” and “P.Off.” This field is optional.
- C.Check: Write the letter “O” when all of the contents in “ChemName,” “C.ID,” and “C.Off” shown in Fig. 2 are correctly annotated and write the letter “X” when any of them are incorrectly annotated.
- C.Note: Leave comments about incorrectly annotated contents in “ChemName,” “C.ID,” and “C.Off.” This field is optional.
- Label: Write “POS” or “NEG” according to the relationship type between a plant, colored in red, and a chemical, colored in green, in the “Sentence” column. “POS” indicates that a plant includes a chemical while “NEG” means that there is no positive relationship between them.
- Weak Trigger: Write trigger terms, which represent the positive relationship between a plant and a chemical, in as broad a range as possible. For example, the weak trigger in the first corpus unit in Fig. 2 can be “were the highest in,” whereas annotators write “NA” when the “Label” column is denoted as “NEG.” This field is required only when the sentence contains a positive relationship between plant and chemical. Leave this field empty when the “Label” is annotated as “NEG.”
- Strong Trigger: Unlike the weak trigger, the strong trigger should be the single word regarding by the annotators as the most meaningful word that represents the positive relationship between a plant and a chemical. For example, the strong trigger in the first corpus unit in Fig. 2 can be “in,” whereas annotators write “NA” when the “Label” column is denoted as “NEG.” This field is required only when the sentence contains a positive relationship between plant and chemical. Leave this field empty when the “Label” is annotated as “NEG.”

After all annotators completed the annotation task, the main annotator collected annotation results to classify agreements or disagreements.

### Constructing a rule development corpus and a rule-based model

We developed a rule-based model to extract plant–chemical relationships from articles. For this task, the main annotator additionally constructed “a rule development corpus,” which is not a duplicate of the primary corpus described in the Annotating the corpus subsection. Then, the performance of the rule-based model was tested against the primary corpus.

**Fig. 3:**
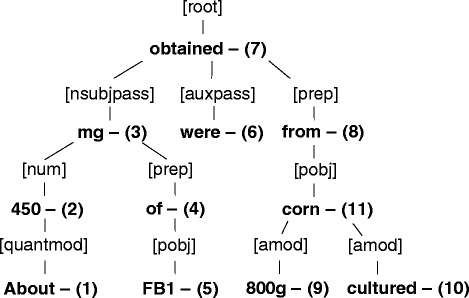
An example of the result of the Stanford Dependency Parser. A tree represents dependency types of each token when applying the Stanford Dependency Parser to the following sentence: “About 450 mg of FB1 were obtained from 800 g cultured corn.” In the dependency parse tree, words in square brackets express dependency types of the linked tokens below. For instance, [nsubjpass] means a passive nominal subject, and it is a noun phrase that is the syntactic subject of a passive clause, “about 450 mg of FB1”. Detailed descriptions of the various dependency types are available in the Stanford Typed Dependencies manual provided by the Stanford NLP

To build the rule development corpus, the main annotator performed the same procedures described in the Sentence collection and preprocessing section and followed the annotation guidelines. The rule development corpus was then used to generate key rules for the model. To infer general grammar rules, we analyzed dependency parse trees (Figs. 3), which were obtained by applying the Stanford Dependency Parser to corpus units in the rule development corpus.

For example, the tree in Fig. 3 shows the dependency structure of a passive sentence, “About 450 mg of FB1 was obtained from 800 g cultured corn.” The noun phrase, “About 450 mg of FB1,” is connected with “nsubjpass” type, which is a passive nominal subject of a passive clause. The noun phrase, “800 g cultured corn,” is connected with “pobj” type, which is an object of a preposition. Then, we can observe that there is a positive relationship between the noun phrase containing a plant name (“corn”) and the noun phrase containing a chemical name (“FB1”) through the trigger, “were obtained from.” Based on such observations, we define a verbal trigger rule when the trigger type is a transitive form, which is defined below. For new inputs that may have a similar dependency structure with Fig. 3, the rule-based model compares the dependency tree of the new inputs with the verbal trigger rule by checking the following: (i) if there is a noun phrase containing a chemical name, which is connected with “nsubjpass” type; (ii) if there is a noun phrase containing a plant name, which is connected with “pobj” type that is an object of a preposition; and (iii) if the root term “obtained,” which is linked with “nsubjpass” and “prep,” belongs to one of trigger words defined in our trigger dictionary.

Based on observing dependency parse trees of the rule development corpus like the example above, our rule-based model contains six types of rules: (i) a verbal trigger rule; (ii) a prepositional trigger rule; (iii) a relative trigger rule; (iv) an apposition trigger rule; (v) a copula trigger rule; and (vi) a compound noun trigger rule. To clarify the rules, we constructed rule specifications that explain the rules in detail (Table 1). Here, we review each of the rule specifications. Note that *NP*_0_ means a noun phrase containing a plant name and *NP*_1_ specifies a noun phrase in which a chemical name appears.

**Table 1:** The rule specifications

Rule specification type	#	Rule structure	Trigger word form	Constraint	Example (PMID)
Verbal trigger rule	1	*N* *P*_0_ *V*_*tr*_ *N* *P*_1_	Transitive verb (active from)	NA	*[* ***Pomegranate*** *derived from the tree Punica granatum] [* ***contains***] [***anthocyanins*** *]. (PMID: 15493960)*
	2	*N* *P*_1_ *V*_*tr*_ *PP* *N* *P*_0_	Transitive verb (passive from)	Any preposition between *V*_*tr*_ and *N* *P*_0_	*[About 450 mg of * ***FB1*** *] [* ***were obtained*** *] [* ***from*** ***] [800g cultured*** ***corn*** *]. (PMID: 23605447)*
	3	*N* *P*_0_ *V*_*tr*_ *PP* *N* *P*_1_	Intransitive verb	Any preposition between *V*_*tr*_ and *N* *P*_1_	*[The volatile oil (2-3 %) of* ***ginger*** *] [* ***consists*** *] [* ***of*** *] mainly [mono and* ***sesquiterpenes*** *]. (PMID: 17637489)*
Preposition trigger rule	1	*N* *P*_0_ *P* *P*_*tr*_ *N* *P*_1_	Preposition	NA	*[* ***switchgrass*** *] [* ***as*** *] [a sole* ***carbon*** *(C) source]. (PMID: 22354956)*
	2	*N* *P*_1_ *P* *P*_*tr*_ *N* *P*_0_	Preposition	NA	*[* ***Saponins*** *] [* ***from*** *] [the flowers of* ***Panax notoginseng*** *]. (PMID: 20518315)*
Relative trigger rule	1	*N* *P*_1_ *R*_*tr*_ *PP* *N* *P*_0_	Past participle form	Any preposition between *R*_*tr*_ and *N* *P*_0_	*[* ***Anthocyanins*** *] [* ***isolated*** *] [* ***from*** *] [* ***black soybean*** *seed coat]. (PMID: 16457818)*
	2	*N* *P*_0_ *R*_*tr*_ (*PP*) *N* *P*_1_	Gerund form	When the trigger word (*R*_*tr*_) is “consisting,” preposition (*PP*), “of,” should be followed by *R*_*tr*_.	*With [thermally degraded* ***Feverfw*** * powder] [* ***containing*** *] [less contents of * ***parthenolide*** *] no built-up antiserotonergic responses were observed after one month. (PMID: 11603284)*
Apposition trigger rule	1	*N* *P*_0_ *A* *P*_*tr*_ *N* *P*_1_	Apposition form (e.g. comma)	The token distance between *N* *P*_0_ and *N* *P*_1_ should be within ten.	*Whereas that in PD is [* ***soybean oil*** *][* ***,*** *] [a source of unsaturated * ***fatty acids*** *]. (PMID: 19932903*
	2	*N* *P*_1_ *A* *P*_*tr*_ *N* *P*_0_	Apposition form (e.g. comma)	The token distance between *N* *P*_0_ and *N* *P*_1_ should be within ten.	*[* ***Delta9-tetrahydrocannabinol*** * (THC)][* ***,*** *] [the major active component of * ***marijuana*** *]. (PMID: 9129126)*
Copula trigger rule	1	*N* *P*_0_ *C*_*tr*_ *N* *P*_1_	Be verb form	The token distance between *N* *P*_0_ and *N* *P*_1_ should be within ten.	*[* ***Haematococcus pluvialis*** *] [* ***is*** *] [one of the potent organisms for production of * ***astaxanthin*** *]. (PMID: 23605447)*
	2	*N* *P*_1_ *C*_*tr*_ *N* *P*_0_	Be verb form	The token distance between *N* *P*_0_ and *N* *P*_1_ should be within ten.	*[The* ***calcium*** * contents] [* ***were*** *] the highest in [the * ***papaya*** *]. (PMID: 21695915)*
Compound noun trigger rule	1	*N* *P*_0_ *C* *N*_*tr*_ *N* *P*_1_	White space	NA	*To study the protective effect of [* ***panax notoginseng*** *] [* ***saponins*** * (PNS)]. (PMID: 19317166)*

The rule-based model consists of six types of rules. The first column shows the specification name. Each specification has more than one rule structure shown in the third column. In the rule structure, *N* *P*_0_ means the noun phrase containing a plant name, and *N* *P*_1_ represents the noun phrase in which a chemical name appears. The component marked with “tr” represents a trigger word described in the fourth column. We also defined several constraints if necessary

- A verbal trigger rule: As shown in Table 1, the specification for the verbal trigger has three types according to the trigger type. The first rule structure is defined as *NP*_0_*V*_*tr*_*NP*_1_, where the trigger type of *V*_*tr*_ is a transitive active form such as “contain” and “include.” The second rule structure consists of *NP*_1_*V*_*tr*_*PPNP*_0_. In this case, the trigger type is a transitive passive form such as “be contained” and “be extracted.” Thus, a preposition denoted as “PP” is required to be located between *V*_*tr*_ and *NP*_0_, which is the constraint. In the third rule structure, *V*_*tr*_ should be an intransitive verb such as “consist.” Therefore, any preposition such as “of” should be placed next to *V*_*tr*_ in the rule structure, *NP*_0_*V*_*tr*_*PPNP*_1_.
- A prepositional trigger rule: The rule structure has the following two cases: *NP*_0_*PP*_*tr*_*NP*_1_ and *NP*_1_*PP*_*tr*_*NP*_0_. Both *NP*_0_ and *NP*_1_ are allowed to be located on the left and right side of the trigger. Any preposition can be placed in *PP*_*tr*_.
- A relative trigger rule: This consists of two types of specifications according to the trigger type. The first rule structure is defined as *NP*_1_*R*_*tr*_*PPNP*_0_, where *R*_*tr*_ is the past participle form such as “isolated” and “extracted,” and any preposition must be located between *R*_*tr*_ and *NP*_0_. The second rule structure is described as *NP*_0_*R*_*tr*_ (*PP*) *NP*_1_, where the trigger type *R*_*tr*_ is a gerund form such as “containing” and “having.” Note that, in this case, the preposition such as “of” should be located between *R*_*tr*_ and *NP*_1_ when the trigger word is the gerund form of the intransitive verb such as “consisting.”
- An apposition trigger rule: The rule structure has the following two cases: *NP*_0_*AP*_*tr*_*NP*_1_ and *NP*_1_*AP*_*tr*_*NP*_0_. In this specification, triggers can be any token whose dependency type is apposition. For example, a comma between *NP*_0_ and *NP*_1_ can indicate that *NP*_0_ is in apposition with *NP*_1_ or vice versa. Furthermore, we simply set a constraint that a token distance between *NP*_0_ and *NP*_1_ is less than ten to avoid the case that does not represent a relationship between them.
- A copula trigger rule: The rule structure has the following two cases: *NP*_0_*C*_*tr*_*NP*_1_ and *NP*_1_*C*_*tr*_*NP*_0_. In this specification, a trigger “ *C*_*tr*_” can be any verb regardless of tense if its dependency type is copula. To reduce false positives, we also set a simple constraint that there must be a token distance between *NP*_0_ and *NP*_1_ of less than ten.
- A compound noun trigger rule: The rule structure is *NP*_0_*CN*_*tr*_*NP*_1_, where the trigger *CN*_*tr*_ is a single white space between *NP*_0_ and *NP*_1_. For example, “Aloe emodin” indicates that emodin is one of the ingredients in aloe.

We manually created a list of trigger words for each of the six types of rules that express a relationship between plant and chemical (Table 2). The list of trigger words is also available to download at the corpus web site.

**Table 2:** Trigger words used in the rule-based model. The table shows trigger words selected for the six predefined rules in the model

Trigger type	Trigger word form	Trigger words
Verbal trigger (*V*_*tr*_)	Active form	contain, contains, contained
		have, has, had
		involve, involves, involved
		incorporate, incorporates, incorporated
		possess, possesses, possessed
		encompass, encompasses, encompassed
		subsume, subsumes, subsumed
		comprise, comprises, comprised
		embody, embodies, embodied
		embrace, embraces, embraced
		include, includes, included
		cover, covers, covered
		compose, composes, composed
		originate, originates, originated
		produce, produces, produced
		derive, derives, derived
		accumulate, accumulates, accumulated
		release, releases, released
	Passive form	contained, involved, incorporated, possessed,
		encompassed, subsumed, comprised, embodied,
		embraced, included, covered, composed, produced,
		originated, derived, accumulated, released, isolated,
		extracted, separated, detached, split, segregated,
		obtained, found, gained, discovered, uncovered, identified
	Intransitive form	consist, consists
Prepositional trigger (*P* *P*_*tr*_)	Preposition	any token whose dependency type is “prep”
Relative trigger (*R*_*tr*_)	Past participle form	*see trigger words in the passive form section above (same as passive form)*
	Gerund form	containing, involving, incorporating, possessing,
		encompassing, subsuming, comprising,
		embracing, including, covering, composing,
		embodying, producing, originating, deriving,
		accumulating, releasing, having, consisting
Apposition trigger (*A* *P*_*tr*_)	Apposition form	any token whose dependency type is “appos”
Copula trigger (*C*_*tr*_)	Copula form	any token whose dependency type is “cop”
Compound noun trigger (*C*_*tr*_)	Compound noun form	strings that are made up of plant and chemical names together (*e.g. panax ginseng saponin*)

### Overall process for extracting plant–chemical relationships

**Fig. 4:**
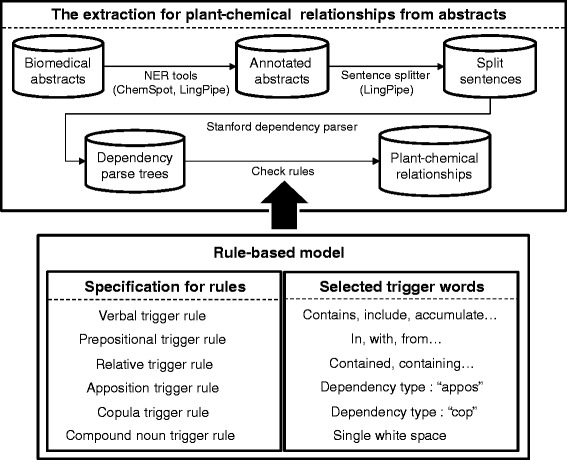
The overall method for extracting plant–chemical relationships from abstracts. To extract plant–chemical relationships from biomedical articles, our model annotates plant and chemical names in the articles using LingPipe and ChemSpot, respectively. Then, the system splits annotated abstracts into sentences using the LingPipe sentence splitter to apply the Stanford Dependency Parser, which provides dependency parse trees for each sentence. Finally, the rule-based model checks the grammar structure of the dependency parse trees and the trigger words in sentences to extract relationships

In Fig. 4, we describe the overall process of the rule-based model when new texts are given to the system. When biomedical abstracts are input to the model, for example, NER tools including ChemSpot [31] and the LingPipe [30] dictionary chunker are applied to annotate plant and chemical names in the new texts, respectively. Then, annotated abstracts are split into sentences using the LingPipe sentence splitter. Next, the Stanford Dependency Parser is applied to each split sentence to obtain dependency parse trees (Fig. 3). The dependency parse trees are then used to check whether the structure of each of the dependency parse trees matches one of the rules defined in our model (Table 1) and also whether there is a trigger word that belongs to our list (Table 2). Finally, our system predicts a class label that represents the relationship type (positive or negative) between the identified plants and chemicals.

## Results

**Fig. 5:**
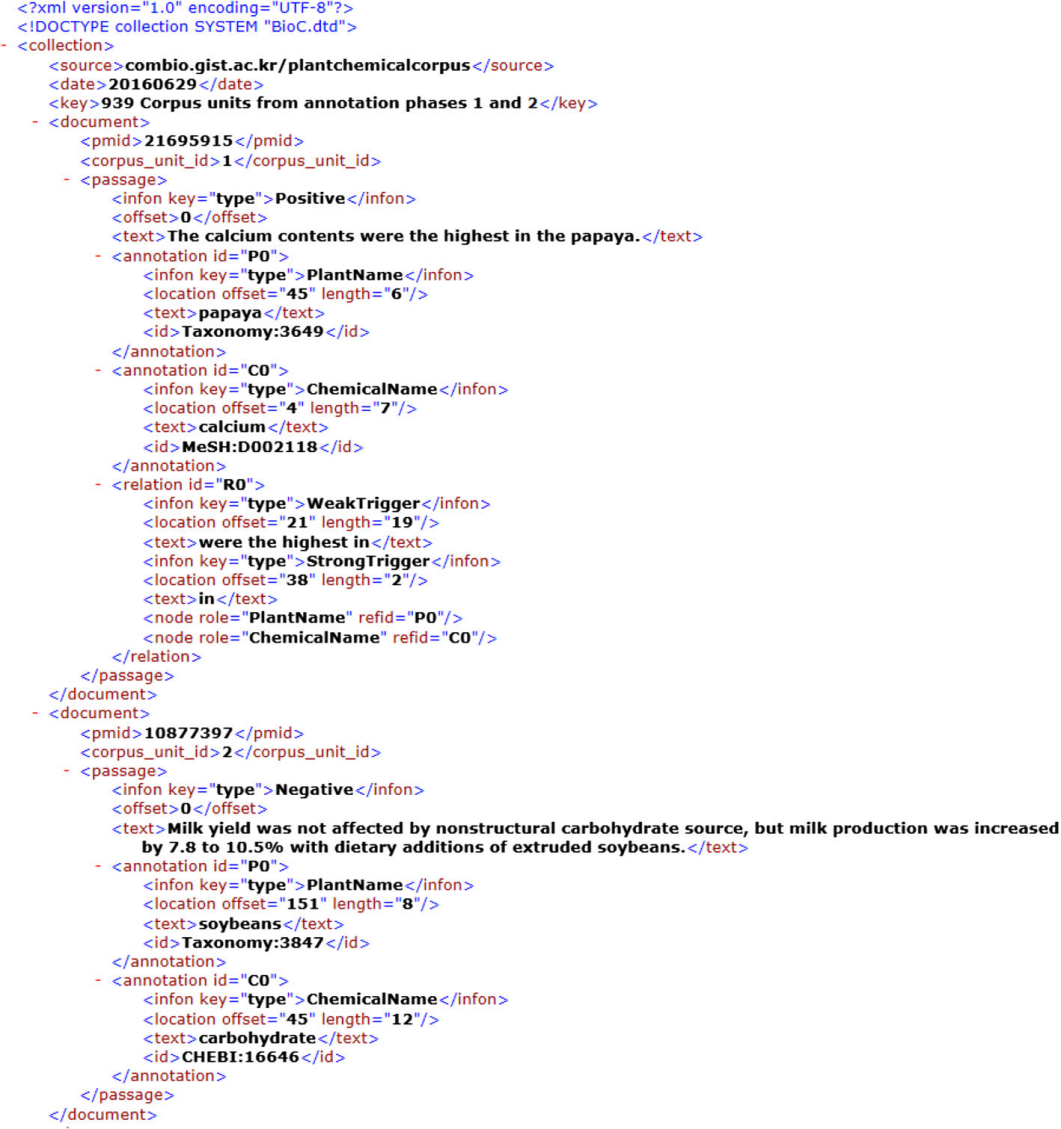
Example of our corpus units in BioC XML format. The corpus contains information about gold-standard sentences with annotated plant and chemical names along with their IDs, locations of entity names, trigger terms only for positive relationships, and class labels (positives or negatives). The corpus data we provide in our web site are currently divided into three types: (i) 939 primary corpus units from annotation phases 1 and 2; (ii) 68 later-annotation corpus units after harmonizing disagreements; and (iii) 102 rule development corpus units

By applying the steps in the “Sentence collection and preprocessing” subsection, we accumulated a total of 1,043 candidate corpus units from 382 candidate sentences, which were obtained from 262 abstracts. The candidate corpus units were divided into phase 1, containing 642 corpus units, and phase 2, containing 401 corpus units, and then annotators in Group 1 and Group 2 annotated the phase 1 units and phase 2 units, respectively. As a result, 939 corpus units out of 1,043 candidate corpus units were agreed upon by the annotators. The remaining 104 corpus units were disagreed upon. The annotators discussed these disagreement units in order to reach consensus about the annotation of entities, relations, and triggers. After the discussion, 1,007 corpus units consisting of 550 positive relationships and 457 negative relationships were finally obtained. The corpus includes 267 plant names and 475 chemical names. For easy access and use, we converted the corpus into BioC XML format (Fig. 5). The corpus provides information about the gold-standard sentences with annotated plant and chemical names along with their IDs, locations of entity names, weak/strong triggers only for positive relationships, and class labels indicating whether the sentence includes a positive or negative relationship.

### Inter-annotator agreement (IAA)

To assess the accuracy of the corpus, we calculated the IAA scores using simple percent agreement and Cohen’s kappa statistic. The simple percent agreement is calculated as follows: [*agreements*/(*agreements*+*disagreements*)]×100 *%*. Cohen’s kappa is the most frequently used method for measuring the overall agreement between two annotators, and it is generally regarded as a more robust measurement than the simple agreement calculation. According to [32], kappa values within the range 61 to 80 % are considered as “substantial” agreement, and values between 81 and 99 % constitute “almost perfect” agreement.

Tables 3 and 4 show overall IAA scores for annotations. In Table 3, using the simple percent agreement, the overall agreement scores were 99.6 and 94.8 % for the annotated entities and triggers, respectively. Although entity names for plants and chemicals were pre-annotated using NER tools and checked by the main annotator, annotators additionally checked whether pre-annotated entity names, their IDs, and locations of entity names were correct in case of any remaining NER errors. When the annotators agreed on entity names, their IDs, and locations of entity names, it is considered as an agreement between annotators. We also checked whether annotators selected the same weak and strong triggers explaining the positive relationship for a given plant–chemical pair. In addition, we measured the degree of agreements for annotations of the relationship between plant and chemical that are positive or negative. When the 1,043 candidate corpus units from 382 candidate sentences were given to annotators, 939 out of 1043 annotated relationship labels were agreed upon by the annotators. Using the Cohen’s kappa statistic, the overall IAA score was 79.8 % for the annotated relationship labels, which can be considered “substantial” agreement according to [32]. Table 4 shows the IAA scores for the case where the same annotation indicates that annotators are in agreement on both entities and relationship labels for each corpus unit. The overall IAA agreement score for this case was 78.9 % using the Cohen’s kappa statistic.

**Table 3:** Statistics and IAA scores for entities, relation labels, and triggers. The IAA scores for entities and relation labels were calculated using a simple percent agreement. The IAA score for relation labels was calculated using Cohen’s kappa statistic

Phases	Entity	# of entities	# of agreements for entities (IAA, Simple)	Relation	# of class labels	# of agreements for relation labels (IAA, Kappa)	Trigger	# of triggers	# of agreements for triggers (IAA, Simple)
Phase 1	Plants	642	640 (99.7 %)	Plant–chemical	642	570 (77.6 %)	Weak	284	275 (96.8 %)
	Chemicals	642	636 (99.1 %)				Strong	284	271 (95.4 %)
	Total	1,284	1276 (99.4 %)				Total	568	546 (96.1 %)
Phase 2	Plants	401	401 (100.0 %)	Plant–chemical	401	369 (82.8 %)	Weak	245	234 (95.5 %)
	Chemicals	401	400 (99.8 %)				Strong	245	223 (91.0 %)
	Total	802	801 (99.9 %)				Total	490	457 (93.3 %)
Overall		2,086	2,077 (99.6 %)		1,043	939 (79.8 %)		1,058	1,003 (94.8 %)

**Table 4:** Statistics and IAA scores of annotated corpus units. The number of agreements was counted when annotators agree on both entities and relation labels. The IAA score in this case was calculated using the Cohen’s kappa statistic

Phases	# of corpus units	# of agreements for both entities and relations	IAA score, Kappa
Phase 1	642	566	76.5 %
Phase 2	401	368	82.0 %
Overall	1043	934	78.9 %

### Disagreements

After the annotation tasks, 104 out of 1,043 candidate corpus units were disagreed upon among the annotators due to the following reasons: subjective interpretation of sentences, misunderstanding of the origin of ingredients, and disregarding guidelines. To harmonize opinions among annotators, annotators performed an additional annotation phase for the disagreed-upon corpus units and specified reasons for their decision. Then, the main annotator collected only the discrepancies that were still in disagreement even after the additional annotation phase, and annotators again discussed the discrepancies to reach consensus on the annotation. As a result, we reached agreement on 68 corpus units from 104 disagreements, and they were added to our plant–chemical corpus dataset. Three types of major disagreement cases are introduced in the following.

- **Example 1.** [Allyl isothiocyanate] _chemical_ (AITC) is [a constituent of] _trigger_ several plants of the family [Cruciferae]_plant_ that are commonly used as food.
  - For the corpus unit in Example 1, one annotator assigned a negative relationship due to misinterpreting the meaning of the sentence. Actually, the sentence includes a positive relationship between the plant “Cruciferae” and the chemical “Allyl isothiocyanate” because they are closely linked with the trigger “a constituent of.”
- **Example 2.** 1,3-dichloropropene (1,3-D) was evaluated as a potential alternative for the widely used soil fumigant [methyl bromide] _chemical_ (MeBr) [in] _trigger_ [cucumber (Cucumis sativus Linn.)] _plant_ crops in China.
  - For the corpus unit in Example 2, one annotator interpreted the chemical “methyl bromide” as one of the ingredients originating from the plant “cucumber.” However, the chemical “methyl bromide” is a powerful pesticide for cultivating cucumber crops, not an original component of the plant. As such, misunderstanding of the origin of ingredients can induce disagreement cases.
- **Example 3.** The authors studied the changes in subjective symptoms of menopause in 2016 Hungarian women who had been treated with an [isopropanol] _chemical_ extract [of] _trigger_ [Cimicifuga racemosa] _plant_ (black cohosh).
  - For the corpus unit in Example 3, one annotator annotated it as a positive relationship between the plant “Cimicifuga racemosa” and the chemical “isopropanol.” In fact, as described in the guidelines, the chemical “isopropanol” is used in a common extraction method for deriving active ingredients from plants. In this case, annotations between annotators were different due to disregarding the guidelines.

### Evaluating the rule-based model

We constructed the rule development corpus consisting of 102 corpus units (50 positives and 52 negatives) from 50 sentences, and this corpus is also provided at corpus website. The rule-based model was developed based on the rule development corpus. We measured the performance of the rule-based model using 939 corpus units in the primary corpus. The 939 corpus units that are inputted to the rule-based model already contain annotation information for entity names, triggers, and their locations in the texts. Then, the model performs the following steps for a given input corpus: (i) apply the Stanford Dependency Parser to the corpus units to obtain dependency parse trees; (ii) check whether there are dependency parse trees that are structurally matched to one of rules defined in the model; (iii) seek trigger words from the sentence only if dependency parse trees of corpus units are matched to one of the rules in the previous step; and (iv) if the trigger word is detected, the system recognizes that there is a positive relationship between plant and chemical for the corpus. Finally, we obtained predicted class labels (positive or negative) of 939 corpus units and compared them with the originally annotated class labels.

As a result, as shown in Table 5, the rule-based model achieved an overall F-measure of 68 %. The F-score for phase 1 was relatively lower than that for phase 2 because corpus units in phase 1 included more diverse variations of grammar structures that were not defined in the rule-based model. Of 939 corpus units, we found that there were 117 false positives and 188 false negatives. False negative errors were mostly due to two reasons: (i) the absence of rules in the model and (ii) the absence of trigger words in the model. For instance, consider “tobacco-specific nitrosamines.” In this case, the rule-based model could not identify it as positive because our model did not contain a grammar structure that can represent “plant-specific chemical,” and also, the trigger word (“-specific”) was not defined. In another example, “3-(methylthio)propanal (cooked potato),” a grammar structure that allows a plant–chemical relationship through brackets was not defined in our model. False positive errors were mainly attributed to misunderstanding of semantic meaning in the rule-based model. For example, consider the following phrase: “dichloromethane extract of Feverfew.” In this case, the model predicted it as positive by the prepositional trigger rule. However, dichloromethane is one of the methods for extracting active components from plants but not an ingredient of feverfew. For the next example: “ammonia treatment of rice straw,” the model syntactically predicted it as positive although the semantic meaning is that ammonia was used to store rice straw at high moisture content and to kill weed seeds, indicating that ammonia was not contained in rice straw.

**Table 5:** Evaluation of a rule-based text mining model to extract plant–chemical relationships using the corpus data

Phase	Positives	Negatives	P (%)	R (%)	F (%)
Phase 1	273	297	66.5	56.0	60.8
Phase 2	239	130	81.0	71.6	76.0
Overall	512	427	73.5	63.3	68.0

We performed an additional evaluation of the rule-based model by applying it to 43 randomly selected PubMed abstracts. The abstracts contain a total of 59 co-occurrence sentences containing both plant and chemical names. Two annotators manually annotated 113 corpus units from 59 co-occurrence sentences. When we applied the rule-based model to the 113 newly annotated corpus units, it achieved an F-score of 61.81 % (precision = 62.96 %, recall = 60.71 %). This new corpus contains 28 positive and 85 negative relationships. Because the new corpus was constructed at the abstract level, the ratio of positive and negative sentences is different from the primary corpus in our work, where the numbers of positive and negative relationships are similar. Although it contains more negative relationships, the performance of the rule-based model is similar to that of phase 1 in the primary corpus, showing the rule-based model can be used for a corpus at the abstract level as well as a corpus at the sentence level.

## Discussion

In this study, we constructed a plant–chemical corpus that can facilitate the development of a relationship extraction system for collecting plant–chemical relationships from texts. Thus, we have introduced guidelines for annotating sentences that represent relationships between plants and chemicals and also described how we constructed the corpus. As a result, we have identified a total of 1,007 plant–chemical corpus units from 377 gold-standard sentences that were selected from 245 PubMed abstracts.

In recent NLP studies, machine learning approaches are more dominantly employed and frequently perform better than rule-based approaches for many NLP tasks [33]. However, the rule-based approach has some advantages; for instance, it is easy to incorporate domain knowledge and to fix the cause of errors although it requires extensive manual labor [34]. In addition, in the process of constructing general rules by analyzing sentences, domain knowledge can be accumulated while machine learning approaches usually work as a black box. In our work, with a relatively small number of 102 corpus units in the rule development corpus, we developed the rule-based model for extracting plant–chemical relationships. Its performance achieved an F-measure of 68 % on 939 corpus units. For comparison, we employed the Turku Event Extraction System (TEES) [35], which is a support vector machine based text mining system. Although TEES was originally developed for extracting relationships between genes and biological events, it can be modified for extracting any binary relationship. We trained TEES with the same 102 corpus units included in the rule development corpus. Because TEES requires a development set, the 68 corpus units that are agreed upon among annotators after resolving the disagreements, was used for the development set. We applied 939 corpus units in the primary corpus to the model. This resulted in an F-measure of 25.7 % on the corpus units, which is poor compared to the rule-based model. This is because the training set was small. Hence, we again trained and evaluated the TEES model using a ten-fold cross validation on a larger set of 1,109 corpus units, including the following: (i) 939 corpus units from the primary corpus; (ii) 68 corpus units that are agreed upon among annotators after harmonizing the disagreements; and (iii) 102 rule development corpus units. Note that 798, 200, and 111 corpus units were used as training, development, and test sets, respectively, for the performance measurement in the ten-fold cross validation. As a result, it achieved an F-measure of 77.7 %. In this respect, it is possible to apply our corpus data to various machine learning methods to achieve better performance.

## Conclusions

Our future work is to develop and apply a text mining model to the prediction of plant–chemical relationship in all the abstracts in PubMed based on the corpus developed in this work. This is challenging work because we need to improve NER models and the relationship prediction model by combining the rule-based model and a machine learning approach. It also requires validating the final accuracy of plant–chemical relationships predicted from all abstracts. When this future work is successful, we expect that we can obtain a large number of plant–chemical relationships. With the increasing importance of natural products, knowledge about active compounds in herbs has become significant. Numerous diseases can be treated by herb compounds, and various medicines, cosmetics, and other products have widely used herbal compounds as their main components. Thus, we believe that our research provides an important step related to herbs or natural products in text-mining studies.
